# Five novel globin gene mutations identified in five Chinese families by next‐generation sequencing

**DOI:** 10.1002/mgg3.1835

**Published:** 2021-10-28

**Authors:** Jie Zhang, Meijuan Xie, Zhiyu Peng, Xiaoyan Zhou, Tingting Zhao, Chanchan Jin, Yuanlong Yan, Xiaohong Zeng, Dongmei Li, Yangjia Zhang, Jie Su, Na Feng, Jing He, Xiangmei Yao, Tao Lv, Baosheng Zhu

**Affiliations:** ^1^ Department of Medical Genetics Yunnan Provincial Key Laboratory for Birth Defects and Genetic Diseases The First People’s Hospital of Yunnan Province Kunming Yunnan China; ^2^ Department of Obstetrics and Gynecology Yunnan Provincial Clinical Research Center for Birth Defects and Rare Diseases The First People’s Hospital of Yunnan Province Kunming Yunnan China; ^3^ Affiliated Hospital of Kunming University of Science and Technology Kunming Yunnan China; ^4^ Department of Hematology The First People’s Hospital of Yunnan Province Kunming Yunnan China; ^5^ BGI Genomics BGI‐Shenzhen Shenzhen Guangdong China

**Keywords:** bioinformatics analysis, next‐generation sequencing, pathogenicity, thalassemia

## Abstract

**Background:**

Thalassemia is one of the most common inherited diseases worldwide. This report presents three novel cases of α‐thalassemia and two novel cases of β‐thalassemia caused by five different mutations in the globin gene.

**Methods:**

Next‐generation sequencing (NGS) was used to identify novel α‐ and β‐thalassemia in five individuals, which was confirmed by Sanger sequencing of the globin gene. Hematological parameters were determined by an automated cell counter, and hemoglobin electrophoresis was carried out by a capillary electrophoresis system, respectively. The isoelectric point (pI), molecular weight, and conservation for the mutations were described by the Internet software programs. The pathogenicity for globin mutations was analyzed by bioinformatics analysis and relative quantitative analysis.

**Results:**

NGS revealed five novel cases of α‐ and β‐thalassemia: HBA2:c.245C>T, HBA2:c.95+11_95+34delCTCCCCTGCTCCGACCCGGGCTCC, HBA2:c.54delC, HBB:c.373C>A, and HBB:c.40G>A. The clinical implications of these mutations were described. Computational predictions were made for pI, amino acid conservation, and pathogenicity of the missense mutation. Relative quantitative data of the α‐globin mRNA were analyzed.

**Conclusion:**

Five novel globin mutations were identified in the populations of China, and those mutations were analyzed to provide a mechanistic view for their pathogenicity. These analyzed results improve genetic diagnostics for thalassemia, which can improve screening programs for thalassemia and prenatal diagnosis for Chinese population.

## Graphical abstract

In this study, we identified five novel mutations in the five families: HBA2:c.245C>T, HBA2:c.54delC, HBA2:c.95+11_95+34delCTCCCCTGCTCCGACCCGGGCTCC, HBB:c.373C>A, and HBB:c.40G>A. The clinical implications of these mutations were described. Computational predictions were made for the isoelectric point, amino acid conservation, and pathogenicity of the missense mutation.

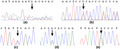

## 1 INTRODUCTION

Thalassemia is a prevalent inherited disease, which includes α‐ and β‐thalassemia (HBA1, OMIM#141800; HBA2, OMIM#141850; HBB, OMIM#603902), characterized by abnormal globin synthesis. It is widely prevalent in Southern China, especially in province of Guangdong, Guangxi, Hainan, and Yunnan (Lai et al., 2017; Yao et al., 2014). Currently, more than 1,300 different mutations of the α‐ and β‐globin gene have been reported in the HbVar database (http://globin.bx.psu.edu/cgi‐bin/hbvar). Specifically, in China, more than 100 different α‐ and β‐globin gene mutations have been documented, while the number is expected to continue growing. The incidence of thalassemia in Southern China has been reported to range from 9.70% to 50.15% (Shang et al., 2017).

Usually, thalassemia screening relies on hemoglobin electrophoresis and routine hematology testing (Fathi et al., 2019). Thalassemia trait usually exhibits abnormal levels of Hb A_2_ or hypochromic microcytic. However, a normal Hb A_2_ level and routine blood parameters cannot rule out one carrying thalassemia since there are asymptomatic carriers (Wu & Li, 2016). Next‐generation sequencing (NGS), as an accurate and rapid detection method for monogenic disease, can produce a large set of data to determine gene sequences and to evaluate possible genetic risks. With a high‐throughput sequencing for overall length gene sequence of α‐ and β‐globin gene, NGS is an accurate molecular diagnosis method for α‐ and β‐globin mutation detection (He et al., 2017; Shang et al., 2017). NGS now has been widely used for molecular screening of thalassemia and prenatal diagnosis in Southern China since 2016 (He et al., 2017).

The present report is a description of five novel mutations of α‐ and β‐thalassemia identified by NGS. The combined use of bioinformatics software analysis and clinical data paved the way for a thorough evaluation of the clinical features and functions of these five mutations. Analytical methods and information about these novel mutations are useful for genetic counseling and programs of thalassemia prevention & control.

## 2 MATERIALS AND METHODS

### 2.1 Ethical compliance

Five individuals from different families were referred for prepregnancy physical examination at the First People's Hospital of Yunnan Province. Gathering of relevant information, obtaining of participants’ consent, and protocols for this study were approved by the Medical Ethics Committee at the First People's Hospital of Yunnan Province.

### 2.2 Genetic sequencing and hematological analysis

Genomic DNA was extracted from peripheral venous blood. These five individuals were screened for α‐ and β‐globin mutation by NGS as previously reported (He et al., 2017). NGS targets three globin genes (*HBA1*, NG_000006.1; *HBA2*, NG_000006.1; *HBB*, NG_000007.3), including the most common copy‐number variations and all point mutations on α‐ and β‐globin genes. Sanger sequencing of the α‐ and β‐globin gene was verified for the five probands and their family members, using a method reported previously (Zhang et al., 2015). The hematological parameters of the five probands and their family members were analyzed by an automated cell counter (Sysmex). Hemoglobin electrophoresis was carried out by a capillary electrophoresis (CE) system (Sebia).

### 2.3 Bioinformatics analysis of α‐ and β‐thalassemia mutations

The missense mutations were analyzed using bioinformatics analysis. The isoelectric points (pI) and molecular weights were analyzed using the Isoelectric Point Calculator (IPC) (http://isoelectric.ovh.org/; Kozlowski, 2016). The amino acids in the mutation locus were examined for evolutionary conservation across 10 randomly selected species.

The pathogenicity of the β‐globin variants was assessed using the HumDiv‐trained model in Polyphen (http://genetics.bwh.harvard.edu/pph2/) and SIFT (https://sift.bii.a‐star.edu.sg/; Li et al., 2020; Liu et al., 2020). For point deletion mutation, the effect of the mutation on resulting peptide was evaluated using mutalyzer (https://mutalyzer.nl).

### 2.4 RNA analysis of α‐thalassemia mutations

To further analyze the pathogenicity of two cases of α‐thalassemia, the α‐globin expression levels (α/β mRNA expression level ratio) were measured by relative quantitative reverse‐transcript PCR technology. Total cellular RNA was extracted using Trizol reagent. And complementary DNA (cDNA) synthesis was performed using the First‐Strand cDNA Synthesis Kit. The relative standard curve method was used to measure the relative amounts of α‐globin mRNA in the patients and normal control. Primers and reaction conditions used in this study were described previously (Mo et al.; 2005).

## 3 RESULTS

### 3.1 Study subjects and carrier screened by NGS and Sanger sequencing

Cumulatively, five cases of novel α‐ and β‐thalassemia were identified by NGS: HBA2:c.245C>T (Ser>Phe at CD81, S81P), HBA2:c.95+11_95+34delCTCCCCTGCTCCGACCCGGGCTCC, HBA2:c.54delC, HBB:c.373C>A (Pro>Thr at CD124, P124T), and HBB:c.40G>A (Ala>Thr at CD13, A13T).

**FIGURE 1:**
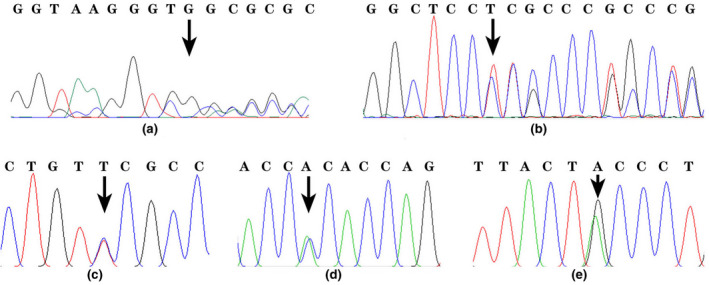
Direct sequencing analysis of the α‐ and β‐globin gene. (a) The arrow points to the “C” nucleotide deletion mutation of the α2‐globin gene. (b) The arrow points to the 24 bp deletion mutation at intron 1 of the α2‐globin gene. (c) The arrow points to the C>T substitution at CD81 of the α2‐globin gene. (d) The arrow points to the C>A substitution of the β‐globin gene. (e) The arrow points to the G>A substitution of the β‐globin gene

The sequences of these five mutations were further confirmed by Sanger sequencing (Figure 1). Family members of those five probands were also sequenced by Sanger sequencing. These five mutations cannot be identified in another group of 1000 patients, so they cannot be considered as gene polymorphism. Those mutations were not reported in HbVar, HGMD, ITHANET, and ClinVar database. Thus, they represented five novel mutations, which, respectively, are designated as Hb Zhaotong (HBA2:c.245C>T), Hb Kunming (HBA2:c.54delC), Hb Qujing (HBA2:c.95+11_95+34delCTCCCCTGCTCCGACCCGGGCTCC), Hb Yuxi (HBB:c.373C>A), and Hb Chuxiong (HBB:c.40G>A). Five mutations are named where five carriers were from Globin genotypes and hematological characteristics of the five families are summarized in Table 1.

**TABLE 1:** The hematological and electrophoretic characterization of five families

Number	Member	HBA Genotype	HBB Genotype	Sex‐Age	RBC (×10^12^/L)	Hb (g/L)	MCV (fL)	MCH (pg)	Hb A (%)	Hb A_2_ (%)	Hb F (%)
Case 1	Proband	α^c^.^245C>T^α/αα	β/β	F‐38	4.56	142	89.7	31.1	97.5	2.50	—
	Father	α^c^.^245C>T^α/αα	β/β	M‐68	4.77	155	97.5	32.5	97.4	2.60	—
	Mother	αα/αα	β/β	F‐62	4.84	149	91.9	30.8	97.3	2.70	—
	Sister	α^c^.^245C>T^α/αα	β/β	F‐36	4.62	139	91.7	31.1	97.3	2.70	—
	Son	αα/αα	β/β	M‐7	5.05	146	83.2	28.9	97.1	2.90	—
Case 2	Proband	α^Hb Qujing^α/αα	β/β	M‐40	5.82	182	90.4	31.3	97.1	2.90	—
	Father	α^Hb Qujing^α/αα	β/β	M‐68	6.17	198	92.7	32.1	97.1	2.90	—
	Mother	αα/αα	β/β	F‐64	4.47	145	94.6	32.4	97.2	2.80	—
	Sister	αα/αα	β/β	M‐44	4.88	146	91.6	29.9	97.4	2.60	—
Case 3	Proband	α^c^.^54delC^α/αα	β/β	F‐26	4.72	125	82.0	26.5	97.6	2.40	—
	Father	α^c^.^54delC^α/αα	β/β	M‐53	5.09	144	88.8	28.3	97.3	2.70	—
	Mother	αα/αα	β/β	F‐50	5.05	152	90.9	30.1	97.6	2.40	—
	Sister	αα/αα	β/β	F‐21	4.73	141	91.5	29.8	97.2	2.80	—
Case 4	Proband	αα/αα	β^c^.^373C>A^/β	F‐27	5.30	162	91.4	30.3	97.0	3.00	—
	Father	αα/αα	β/β	M‐50	5.17	167	82.4	31.5	97.3	2.70	—
	Mother	αα/αα	β^c^.^373C>A^/β	F‐49	4.60	145	95.7	32.3	97.0	3.00	—
	Brother	αα/αα	β/β	M‐29	5.76	181	88.7	31.4	97.3	2.70	—
Case 5	Proband	αα/αα	β^c^.^40G>A^/β	F‐25	3.42	112	99.1	32.9	97.2	2.80	—
	Mother	αα/αα	β/β	F‐51	4.47	132	92.6	29.5	97.1	2.90	—
	Sister	αα/αα	β/β	F‐30	4.74	146	88.8	30.8	97.2	2.80	—

Note

Hb Qujing: HBA2:c.95+11_95+34delCTCCCCTGCTCCGACCCGGGCTCC. Normal values of hematological parameters: RBC (4.00–5.50 × 10^12^/L), Hb (120–160 g/L), MCV (82.0–95.0 fL), MCH (27.0–31.0 pg), Hb A (≥94.5%), Hb A_2_ (2.5%‐3.5%), and Hb F (≤2.0%).

The proband of case 1 was a 38‐year‐old female carrying HBA2:c.245C>T. The patient's blood cell count was unremarkable. DNA sequencing detected the mutation in a heterozygous state in the index patient, her sister and father. Her son and mother did not carry the mutation.

The proband of case 2 was a 40‐year‐old male carrying HBA2:c.95+11_95+34delCTCCCCTGCTCCGACCCGGGCTCC. The same α‐thalassemia mutation was identified in his father. The index patient and his father had similar normal levels of the hematological and electrophoretic parameters.

The proband of case 3 was a 26‐year‐old female carrying HBA2:c.54delC. The carrier had slight α‐thalassemia phenotypes with hypochromic and microcytic erythrocytes. This mutation was found in her father but not detected in her mother and sister.

The proband 4 presented the HBB:c.373C>A mutation in a heterozygote state, leading to an amino acid change (Pro>Thr). The carrier had normal hematological parameters. Her mother was also shown to be a carrier of the HBB:c.373C>A mutation, whereas her father and her brother were normal individuals.

Concerning the proband 5 of HBB:c.40G>A mutation, the point mutation c.40G>A in CD13 corresponded to an Ala>Thr substitution. She had normal CE patterns and normal blood parameters. Her father could not attend to any testing due to serious illness. Only the proband was shown to be a carrier of the HBB:c.40G>A mutation in her family,

### 3.2 Bioinformatics analysis of α‐ and β‐thalassemia mutations

**FIGURE 2:**
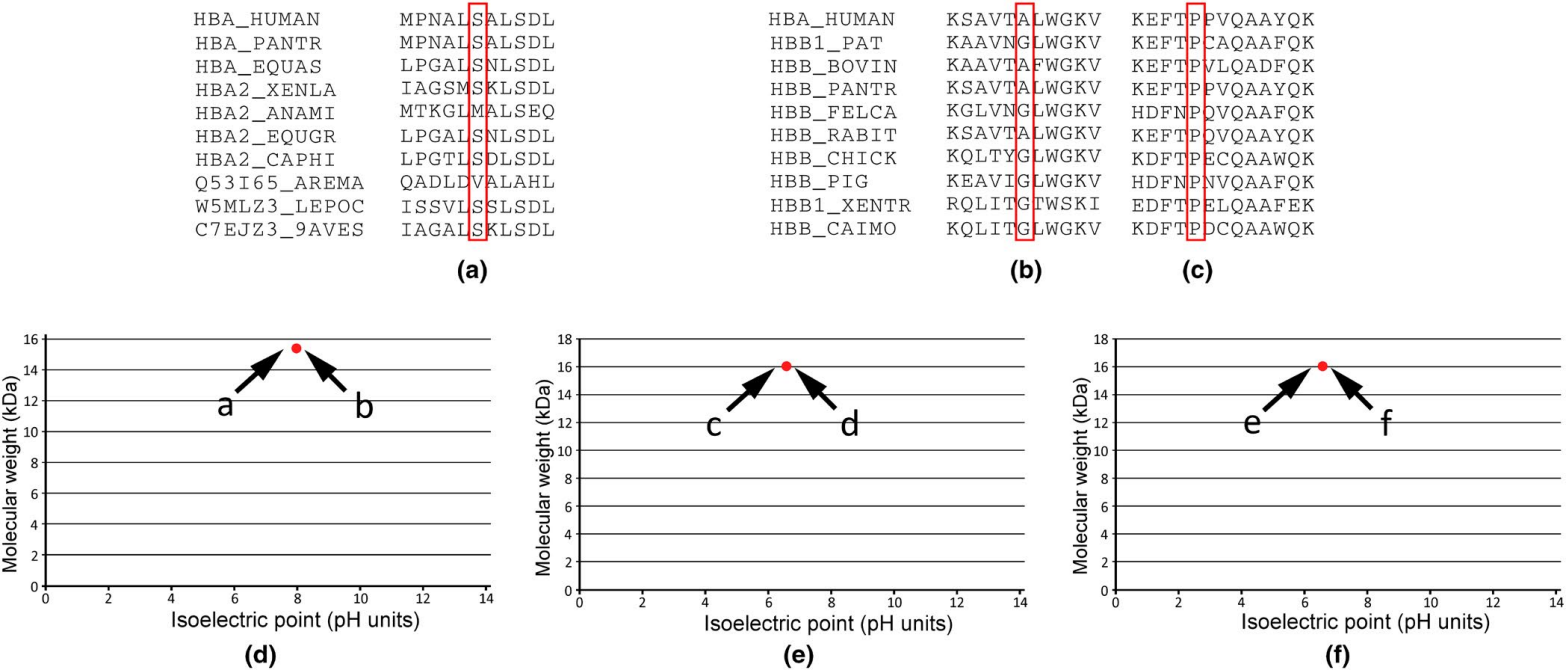
Bioinformatics analysis. (a) Genetic comparison of Ser81 of the α2‐globin in 10 vertebrate species, where Ser81 is conserved in eight species. (b) The Ala13 of the β‐globin was only conserved among five species. (c) The Pro124 of the β‐globin was conserved among all the 10 species. (d) For HBA2:c.245C>T mutation, the pI and molecular weight of the mutant (a, pI 8.12, molecular weight 15 kDa) compared to the wild type protein (b, pI 8.12, molecular weight 15 kDa). And no changes in pIs and molecular weights were observed for HBB:c.373C>A (e) and HBB:c.40G>A mutations (f)

For three missense mutations (HBA2:c.245C>T, HBB:c.373C>A, and HBB:c.40G>A), their molecular effect on the globin function and repercussions on the structure and function of the protein were analyzed by online bioinformatics analysis. Ser at CD81 (Ser81, HBA2:c.245C>T) was conserved in eight species, but varied in another two species (Figure 2a). The conservation of Ala residue in CD13 (Ala13, HBB:c.40G>A) was variable (Figure 2b), whereas the Pro residue in CD124 (Pro124, HBB:c.373C>A) was conserved across 10 species (Figure 2c). No changes in pIs and molecular weights were observed for HBA2:c.245C>T, HBB:c.373C>A, and HBB:c.40G>A mutations (Figure 2d–f).

For two β‐thalassemia mutations (HBB:c.373C>A and HBB:c.40G>A), two different software (Polyphen and SIFT) were used for the prediction of pathogenicity. Results from running those software indicated that HBB:c.373C>A mutation was deleterious, while HBB:c.40G>A mutation was benign. For α‐thalassemia (HBA2:c.53delC), the nucleotide frameshift would result in a shortened α‐globin chain with a total of 48 amino acids according to mutalyzer software.

### 3.3 RNA analysis of α‐thalassemia mutations

The α/β mRNA expression level ratio was measured to analyze the functional consequences of two cases of α‐thalassemia. When the α/β mRNA expression level ratio of normal control was defined as 1.0, the α/β mRNA expression level ratio of HBA2:c.53delC was 0.69. This result indicated that the α‐globin mRNA level of the HBA2:c.53delC heterozygotes was decreased when compared with normal people. For HBA2:c.95+11_95+34delCTCCCCTGCTCCGACCCGGGCTCC, the α/β mRNA expression level ratio was 1.1. This result indicated that the α‐globin gene transcription level of this mutation was nearly normal.

## 4 DISCUSSION

Approximately 1815 mutations on the globin loci have been described to date (http://globin.bx.psu.edu/cgi‐bin/hbvar/counter). With the development of diagnostic methods, an increasing number of novel mutations in the globin genes have been identified (Petropoulou et al., 2015; Tsao et al., 2019). Novel globin mutations are continually identified by advanced methods such as NGS, which exhibits high‐quality genotype coverage and cost‐effectiveness. NGS has been widely used for thalassemia screening, especially for rare silent thalassemia carriers who might be missed using routine thalassemia test methods. Although the majority of heterozygotes for silent thalassemia is asymptomatic, there are still cases exhibiting symptoms such as hemolytic anemia, polycythemia, or thalassemia phenotype, especially when other hemoglobinopathies are combined (He et al., 2018; Jiang et al., 2017). Therefore, accurate identification and differential diagnosis of these clinically relevant variants from other variants are of critical significance. In this study, five novel thalassemia mutations were identified using NGS and Sanger sequencing, and their clinical and structural characterization were analyzed in detail.

Among the five novel thalassemia mutations, only the hematological parameter of HBA2:c.54delC heterozygous carrier showed hypochromic microcytic. However, the heterozygous carriers of other four mutations (HBA2:c.95+11_95+34delCTCCCCTGCTCCGACCCGGGCTCC, HBA2:c.245C>T, HBB:c.373C>A, HBB:c.40G>A) were asymptomatic without hematological or electrophoretic change. Therefore, these four novel mutations should be regarded as silent thalassemia mutations, which cannot be screened by routine methods, thus these results further strengthened previous claim that NGS is an more effective screening method for silent thalassemia (He et al., 2017).

There are two copies of α‐globin gene (α2α1) on chromosome 16, therefore each diploid individual carries four copies of α‐globin gene (α2α1/α2α1). Most non‐deletion α‐thalassemia mutations involved only one single α‐globin gene, and these α‐globin mutations generally do not have clinical consequences which can be regarded as silent thalassemia, as the other three normal α‐globin genes can compensate for the mutation (Hamali & Saboor, 2019). Usually, non‐deletion α‐thalassemia heterozygous are silent carriers with normal hematological profiles, except for few mutations such as αα^CS^ (HBA2:c.427T>C) (He et al., 2017).

For the HBA2:c.245C>T (CD81, Ser>Phe) mutation, the neutral amino acid Ser81 was replaced by the neutral amino acid Phe at CD81 (Phe81) of the α2‐globin chain. This mutation did not affect the pI and molecular weight of the protein. Evolutionary analysis of Ser81 revealed that it is comparatively conserved in nature. Three variants have already been described at CD 81 of the α2‐globin gene: Hemoglobin Nigeria (Honig et al., 1980), Hb Wolverhampton (Henderson et al., 2016), and Hb Passy (Lacan et al., 2005). Hemoglobin Nigeria was described in 1966 in Africa and Hb Passy was described in 2005. The heterozygote carrier of Hemoglobin Nigeria did not have hematological changes. However, the heterozygote carrier of Hb Passy was a patient with hypochromic microcytic anemia.

HBA2:c.54delC is a “C” nucleotide deletion mutation in extron 1 of the α2‐globin gene. Nucleotide deletion mutation can lead to the change of coding sequence of the α2‐globin gene. The blood examinations of the proband indicated hypochromic microcytic anemia. Similar results also be reported in other nucleotide deletion mutation in extron 1 of the α2‐globin gene such as HBA2:c.60delG and HBA2:c.94_95delAG (Harteveld et al., 2003; Zhao et al., 2012). And the result of α‐globin expression level ratio also indicated that this mutation should be pathogenic.

HBA2:c.95+11_95+34delCTCCCCTGCTCCGACCCGGGCTCC led to a 24 bp deletion in intron 1 of the α2‐globin gene. This mutation does not affect the coding sequence of the α2‐globin gene, therefore this mutation is not expected to modify the pI, molecular weight, elution pattern in CE, and structure of the α2‐globin chain. Based on the result of the α‐globin gene transcription level, it can be speculated that this novel mutation might not reduce the expression of α2‐globin. And this mutation should be benign.

Regarding the HBB:c.373C>A mutation, the nonpolar hydrophobic amino acid proline (Pro) is replaced by a polar hydrophobic amino acid Thr at CD124 of the β‐globin chain. The change of amino acid P124T did not affect the pI and molecular weight of the mutation; therefore, theoretically, there should be no abnormal band separation from Hb A in the pattern of CE. Evolutionary conversation indicated that this amino acid (Pro124) is conserved in nature. This result suggested that Pro124 might be a critical residue across the species, so that mutation in this position might be detrimental. Two internet software programs (PolyPhen‐2 and SIFT) also predicted that this missense mutation might be a deleterious type, however, the heterozygous carriers of HBB:c.373C>A were asymptomatic without clinical consequences. In the HbVar database, four genotype mutations within CD124 have already been described: Hb Tunis (HBB:c.373C>T) (Koseler et al., 2010), Hb Ty Gard (HBB:c.374C>A) (Wahengbam et al., 2019), Hb Tende (HBB:c.374C>T) (Wajcman et al., 1998), and Hb Khartoum (HBB:c.374C>G) (Hendy et al., 1999). Similar to HBB:c.373C>A, all these mutations were silent β‐thalassemia.

Concerning HBB:c.40G>A mutation, only the proband was shown to be a carrier. The mutation might be a *de novo* mutation or inherited from her father. Hematological parameters and hemoglobin electrophoresis of the carrier did not show the clinical phenotypes of the thalassemia trait. For all the 10 species analyzed for conservation, Ala13 was observed only in four species (Figure 2b), suggesting that this amino acid residue should not be an important biological residue. The pathogenic effects of the missense mutations predicted by Polyphen‐2 and SIFT prediction protocols showed that the mutation was benign. HBB:c.40G>A mutation is the second identified mutation at CD13 of β‐globin worldwide (Djoumessi et al., 1981).

In conclusion, NGS panel was useful to screen novel mutations in populations. The hematological and electrophoretic characterization of five novel thalassemia were described in details. Furthermore, the conservation and pathogenicity of three novel mutations were analyzed by bioinformatics software programs. The present study provided guidelines for future studies and for the diagnosis of thalassemia.

## ETHIC STATEMENT

This study were approved by the Medical Ethics Committee at the First People’s Hospital of Yunnan Province. All patients provided written informed consent.

## ACKNOWLEDGEMENTS

This study was part of a project financed by National Natural Science Foundation of China (81760037, 81860040, 81660022, 81860281); Ten Thousand People Planning Commission of Yunnan province ( YNWRMY‐ 2020‐066); Young and Middle‐aged Academic Leaders Planning Commission of Yunnan province (202105AC160034); and Academic Leader Programme of Health and Family Planning Commission of Yunnan province (D‐2017056).

## CONFLICT OF INTEREST

The author reports no conflict of interest in this work.

## AUTHOR CONTRIBUTION

JZ, MJ, TZ, and CJ Performed the experiments. TL and BZ contributed to project design. JZ wrote the manuscript draft. All authors contributed to data collection and analysis as well as revision and final approval of the published manuscript.
